# Optimizing identification of consensus molecular subtypes in muscle-invasive bladder cancer: a comparison of two sequencing methods and gene sets using FFPE specimens

**DOI:** 10.1186/s12885-023-11016-9

**Published:** 2023-06-04

**Authors:** Florestan J. Koll, Claudia Döring, Csilla Olah, Tibor Szarvas, Jens Köllermann, Benedikt Hoeh, Felix K.-H. Chun, Henning Reis, Peter J. Wild

**Affiliations:** 1 Department of Urology, University Hospital Frankfurt, Goethe University, Theodor-Stern-Kai 7, 60590 Frankfurt Am Main, Germany; 2 Frankfurt Cancer Institute (FCI), University Hospital, Goethe University, Theodor-Stern-Kai 7, 60590 Frankfurt Am Main, Germany; 3 University Cancer Center (UCT) Frankfurt, University Hospital, Goethe University, Theodor-Stern-Kai 7, 60590 Frankfurt Am Main, Germany; 4 Dr. Senckenberg Institute of Pathology, University Hospital Frankfurt, 60590 Frankfurt Am Main, Germany; 5 Department of Urology, University of Duisburg-Essen, Essen, Germany; 6 Department of Urology, Semmelweis University, Budapest, Hungary; 7 Frankfurt Institute for Advanced Studies (FIAS), 60438 Frankfurt Am Main, Germany

**Keywords:** Bladder cancer, Consensus classification, MIBC, Molecular subtypes, Sequencing

## Abstract

**Background:**

Molecular subtypes predict prognosis in muscle-invasive bladder cancer (MIBC) and are explored as predictive markers. To provide a common base for molecular subtyping and facilitate clinical applications, a consensus classification has been developed. However, methods to determine consensus molecular subtypes require validation, particularly when FFPE specimens are used. Here, we aimed to evaluate two gene expression analysis methods on FFPE samples and to compare reduced gene sets to classify tumors into molecular subtypes.

**Methods:**

RNA was isolated from FFPE blocks of 15 MIBC patients. Massive analysis of 3’ cDNA ends (MACE) and the HTG transcriptome panel (HTP) were used to retrieve gene expression. We used normalized, log2-transformed data to call consensus and TCGA subtypes with the consensusMIBC package for R using all available genes, a 68-gene panel (ESSEN1), and a 48-gene panel (ESSEN2).

**Results:**

Fifteen MACE-samples and 14 HTP-samples were available for molecular subtyping. The 14 samples were classified as Ba/Sq in 7 (50%), LumP in 2 (14.3%), LumU in 1 (7.1%), LumNS in 1 (7.1%), stroma-rich in 2 (14.3%) and NE-like in 1 (7.1%) case based on MACE- or HTP-derived transcriptome data. Consensus subtypes were concordant in 71% (10/14) of cases when comparing MACE with HTP data. Four cases with aberrant subtypes had a stroma-rich molecular subtype with either method. The overlap of the molecular consensus subtypes with the reduced ESSEN1 and ESSEN2 panels were 86% and 100%, respectively, with HTP data and 86% with MACE data.

**Conclusion:**

Determination of consensus molecular subtypes of MIBC from FFPE samples is feasible using various RNA sequencing methods. Inconsistent classification mainly involves the stroma-rich molecular subtype, which may be the consequence of sample heterogeneity with (stroma)-cell sampling bias and highlights the limitations of bulk RNA-based subclassification. Classification is still reliable when analysis is reduced to selected genes.

**Supplementary Information:**

The online version contains supplementary material available at 10.1186/s12885-023-11016-9.

## Background

Sequencing techniques have advanced, leading to broad genomic analyses of bladder cancer cohorts and enabling molecular subtyping. Subtyping of muscle-invasive urothelial bladder cancer (MIBC) categorizes heterogenous cancers with similar molecular and biological characteristics, which has significantly contributed to our knowledge in the recent years [1]. Several groups have simultaneously worked on molecular subtyping of different bladder cancer datasets coming to a description of two main types (luminal and basal), that can further be subclassified into 3–10 subtypes [2–8]. Different nomenclatures, definitions, and numbers of molecular subtypes had hindered further prospective validation, and clinical translation until the description of a consensus classification. The molecular consensus classification used pooled mRNA expression profiles of 1750 fresh frozen and formalin-fixed, paraffin-embedded (FFPE) MIBC samples and identified six molecular classes: luminal papillary (LumP), luminal nonspecified (LumNS), luminal unstable (LumU), stroma-rich, basal/squamous (Ba/Sq), and neuroendocrine-like (NE-like) [3].

The aim of classifying cancer in subgroups is to identify tumors that share similar prognosis and response to various therapies. In several reports molecular subtypes have been described as predictors of response to chemotherapy and immunotherapy [5, 9–12]. However, the results are conflicting, and to date, the evidence is insufficient to use molecular subtyping or other gene expression signatures for the treatment decisions in patients with urothelial carcinoma. To facilitate the implementation of molecular subtyping into daily clinical routine, gene sets have been reduced to allow quantification with quantitative RT-qPCR, NanoString or immunohistochemistry panels [13–20].

Many molecular profiling studies and molecular subtyping in The Cancer Genome Atlas (TCGA) are based on fresh frozen tissue, which allows high quality transcriptomic analyses based on RNA sequencing. However, fresh frozen samples are rarely available in clinical practice and for retrospective research projects. The use FFPE tissue is the gold-standard for pathological analyses and long-term storage in hospitals. The paraffin material is usually archived for 10 and more years, allowing correlation with long-term patient outcome. The disadvantage of FFPE tissue is that the RNA is highly degraded by fixation and storage, leading to sequencing artifacts and limiting detection of transcripts [21, 22]. However, advances in sequencing techniques also enable molecular profiling of FFPE specimen [23–25].

In this study, we tested and compared the feasibility of two RNA sequencing methods with FFPE tissues from MIBCs to determine uniform molecular subtyping. In addition, we used two reduced predefined gene sets to determine molecular subtypes and compared results with the comprehensive transcriptome data.

## Methods

### Patients and samples

Tumor samples were provided by the University Cancer Center Frankfurt (UCT). Written informed consent was obtained from all patients. The study was approved by the institutional review boards of the UCT and the ethical committee at the University Hospital Frankfurt (project-number: SUG-6–2018, UCT-53–2021), and conducted according to local and national regulations and to the Declaration of Helsinki. Fifteen FFPE samples from MIBC patients were obtained from the Dr. Senckenberg Institute of Pathology.

### Immunohistochemistry (IHC)

Samples were stained for CK5/6 (Clone: D5/16 B4; Dako /Agilent, Santa Clara, CA, USA) and GATA3 (Clone: L50-823; Cell Marque, Rocklin, CA, USA) as described before [19].

### RNA-isolation

For each RNA isolation, a 1-mm punch was taken from FFPE blocks of an annotated, representative tumor area with at least 50% tumor content. RNA was either extracted using the truXTRAC FFPE total NA Kit (Covaris, Woburn, MA, USA) or by GenXPro GmbH.

### HTG transcriptome panel (HTP)

The mRNA expression was determined using the HTP (HTG Molecular Diagnostics, Tuscon, AZ, USA) as describes before [19, 26]. Briefly, target capture was done by the HTG EdgeSeq chemistry with nuclease protection probes on a 96-well plate. Processed samples were used to set up PCR reactions with specially designed primers, referred to as “tags”. These tags share common sequences that are complementary to 5’-end and 3’-end “wing” sequences of the probes and common adaptors required for cluster generation on an Illumina sequencing platform. In addition, each tag contains a unique barcode that is used for sample identification and multiplexing. The library was prepared using a PCR with OneTaq (New England Biolabs, Ipswich, MA, USA) and EdgeSeq PCR tag primers (HTG Molecular Diagnostics). Sequencing was performed on the Illumina NextSeq 550 system (Illumina, San Diego, CA, USA) in accordance with manufacturer’s recommendations but also including two HTG custom sequencing primers. The sequencing data on mRNA expression of target genes were imported into HTG EdgeSeq Parser software for alignment of FASTQ files to the to the probe list and quantification of the reads. The HTG EdgeSeq Reveal Application was utilized to quality check and normalize data. Gene counts were normalized using CPM and median normalization and log2-transformed for further analysis.

### MACE Seq

Massive Analysis of cDNA (MACE) is a 3’mRNA sequencing method based on the analysis of Illumina reads derived from fragments that originate from 3’ mRNA ends [24, 27]. Samples were prepared by GenXPro GmbH (Frankfurt, Germany) using the MACE-Kit V2 according to the manual of the manufacturer (GenXPro GmbH). RNA was fragmented and polyadenylated mRNA was enriched by poly-A specific reverse transcription, a specific adapter was integrated at the 5’ ends, and the products were amplified by competitive PCR and sequenced on an Illumina NextSeq 500 instrument. Duplicate reads as determined by the implemented unique molecular identifiers (TrueQuant IDs) were removed from the raw dataset. Low quality sequence-bases were removed by the software Cutadapt (https://github.com/marcelm/cutadapt/) and poly(A)-tails were clipped by an in-house Python-Script. The reads were mapped to the human genome (hg38) and transcripts were quantified by HTSeq [28].

### Molecular subtyping

Molecular consensus classes of MIBC were assigned using the consensusMIBC package for R for the nearest-centroid transcriptomic classifier (https://github.com/cit-bioinfo/consensusMIBC), TCGA classes were assigned using the BLCAsubtyping package (https://github.com/cit-bioinfo/BLCAsubtyping) as described by Kamoun et al. [3]. The minimal threshold for best Pearson’s correlation was set to 0.2. Normalized and log2-transformed gene expression values were used. Retrieved data include the consensus class, the Pearson’s correlation coefficient between each sample and each consensus class, the *p*-values associated to the Pearson’s correlation of the samples with the nearest centroid (correlation *p*-value) and the separation level.

For comparison, we used the included example data set of the TCGA bladder cancer cohort, which was created from fresh tumor specimen [2].

We reduced the gene set input according to two proposed panels for bladder cancer subtyping (Table S1 – S2) [16, 20]. The “ESSEN1”-panel is a 68-gene set covering tumor and stromal signatures [16]. The above panel was further optimized and condensed to a set of 48 genes (called “ESSEN2” in the present study) [20].

The heatmap was constructed with the open-source Morpheus software (https://software.broadinstitute.org/morpheus/) using log2-transformed normalized gene expression values. For hierarchical clustering we used Pearson’s correlation metric with complete linkage.

## Results

### Obtained sequencing data used for molecular classification

Characteristics of the patients included in the study are shown in Table 1.

**Table 1:** Characteristics of 15 urothelial muscle-invasive bladder cancer patients included in the paired comparison

**Variable**		**Study cohort (** ***n*** ** = 15)**
**Median age, years (IQR):**		66 (57.5–72.5)
**Gender – no. (%)**	male	12 (80%)
	female	3 (20%)
**pathological tumor stage at cystectomy – no. (%)**	pT2	2 (13%)
	pT3	10 (67%)
	pT4	3 (20%)
**Source of specimen – no. (%)**	TURB	2 (13%)
	Cystectomy	13 (87%)

*IQR* inter quartile range

With MACE, 22,729 of 29,716 transcripts were mapped to HUGO Gene Nomenclature Committee (HGNC) symbols. With the HTP, 19,399 mRNA transcripts were detected. 16,645 genes had the same HGNC symbol, and 6084 and 2754 genes were detected with either MACE or HTP only, respectively. One HTP sample failed quality control (after sequencing) because of minimal expression variability as determined by median log2 for negative control probes. The median time from sample collection (time of surgery) to analysis was 5 years (range 3–10 years); seven samples were < 5 years and eight samples were ≥ 5 years. There was no significant correlation between the age of the samples and the overall degree of separation, nor was the association of the Pearson correlation coefficients of the samples with the nearest centroid (correlation *p*-value), for either method (Figure S1).

### Comparison of classification values between FFPE-sequencing methods and TCGA-data

We used MACE-, HTP- as well as TCGA-transcriptome data to assign molecular consensus subtypes. We analyzed the data and compared the output information. The association of the Pearson’s correlation coefficients of the samples with the nearest centroid (correlation *p*-value) was < 0.0001 for all samples with either method. However, we noticed significantly higher median correlation *p*-values for subtype calls resulting from HTP vs. MACE vs. TCGA data (Figure S2).

**Fig. 1:**
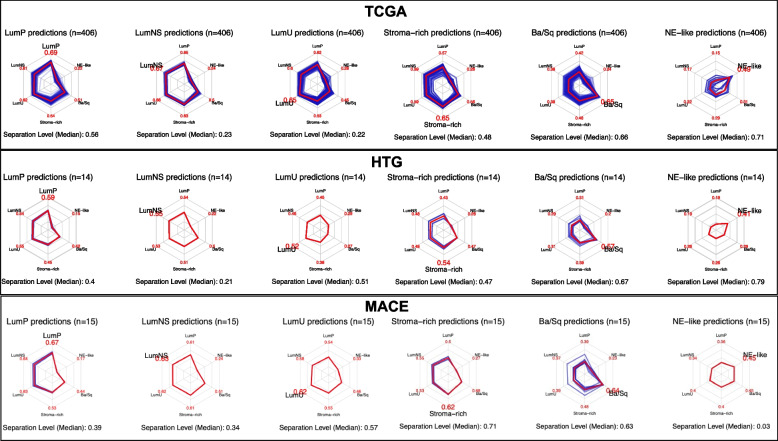
Radar-charts for each consensus class for TCGA, HTP and MACE data. The data are generated from the consensus classifier. Blue lines indicate the Pearson’s correlation coefficient between each sample and each consensus class. Red lines and values indicate the median Pearson’s correlation between samples and each consensus class. The median separation level gives a measure of how a sample is representative of its consensus class and is calculated as follows: (correlation to nearest centroid—correlation to second nearest centroid) / median difference of sample-to-centroid correlation

Figure 1 shows the correlation plots as radar-charts for each consensus class for the 406 TCGA, 14 HTP and 15 MACE samples. Using the MACE data resulted in higher correlation values of the sample to the centroids of the six consensus classes, which was only statistically significant for the Ba/Sq subtype (*p*(Wilcoxon) = 0.0092). With the HTP data, NE-like and Ba/Sq subtype had the highest median separation levels of 0.67 and 0.79, like the TCGA data. Using the MACE data, the stroma-rich subtype had the highest median separation level of 0.71. Based on MACE data, the NE-like subtype had a very low separation level of 0.03. However, this was only based on a single sample. With the HTP and MACE data LumNS subtype and with the TCGA data LumNS and LumU had rather low separation levels, which might be due to other luminal groups.

### Molecular and histological subtypes and IHC-status of patients

**Fig. 2:**
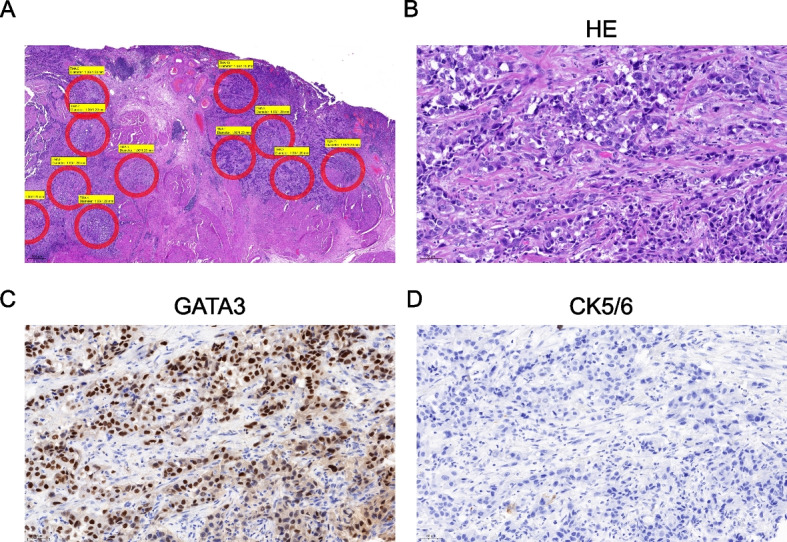
Case 46, which was classified as stroma-rich with the MACE data and LumNS with the HTP data, shown in the HE-overview with tumor annotations for cores for the construction of TMAs and RNA-Isolation (**A**). Further images show HE-staining (**B**), positive GATA3-IHC (**C**) and negative CK5/6-IHC (**D**), 200x

Samples were classified as Ba/Sq in 7 (50%), LumP in 2 (14.3%), LumU in 1 (7.1%), LumNS in 1 (7.1%), NE-like in 1 (7.1%) and stroma-rich in 2 (14.3%) cases with HTP data. And classified as Ba/Sq in 8 (53.3%), LumP in 2 (13.3%), LumU in 1 (6.7%), LumNS in 1 (6.7%), NE-like in 1 (6.7%) and stroma-rich in 2 (13.3%) cases with MACE data (Table 2). Concordance of the consensus classification was 71% (10/14). Two cases (48 and 46) classified as stroma-rich using MACE data were Ba/Sq (48) and LumNS (46) with HTP data. Two cases (49 and 20) classified as stroma-rich with HTP data were Ba/Sq (49) and LumNS (20) with MACE data. Thus, all four discordant cases had a stroma-rich subtype involved with either method. Those cases showed a usual (NOS) histological subtype and no or only low CK5/6 protein expression. An example of one discordant case is shown in Fig. 2. Importantly, LumU and LumP subtypes were concordant in 100% and displayed the expected matching histological subtypes (NOS) and protein expression (CK5/6 negative, GATA3 positive). One NE-like case showed neuroendocrine histological subtype and was negative for CK5/6 and GATA3 IHC. The Ba/Sq was concordant in 86% (6/7) and displayed squamous histological subtype in four cases (57%), NOS in two cases (29%), and lymphoepithelial histological subtype in one case, which has been described to be associated with Ba/Sq molecular subtype [29].

**Table 2:** Molecular subtype, histological subtype and immunohistochemical expression of CK5/6 and GATA3 for each patient

ID	MACE consensus	HTP consensus	MACE TCGA	HTP TCGA	CK5/6 IHC	GATA3 IHC	Histological subtype
41	Ba/Sq	Ba/Sq	Basal squamous	Basal squamous	pos	pos	Squamous
21	LumU	LumU	Luminal	Luminal papillary	neg	pos	NOS
7	LumP	LumP	Luminal papillary	Luminal papillary	neg	pos	NOS
32	Ba/Sq	Ba/Sq	Luminal infiltrated	Basal squamous	pos	pos	Squamous
26	NE-like	NE-like	Neuronal	Neuronal	neg	neg	Neuroendocrine
49	Ba/Sq	Stroma-rich	Basal squamous	Basal squamous	neg	neg	NOS
48	Stroma-rich	Ba/Sq	Luminal infiltrated	Basal squamous	neg	pos	NOS
45	Ba/Sq	Ba/Sq	Basal squamous	Basal squamous	pos	pos	Squamous
44	Ba/Sq	Ba/Sq	Basal squamous	Basal squamous	neg	neg	Lymphoepithelial
8	Ba/Sq	Ba/Sq	Basal squamous	Basal squamous	neg	pos	NOS
42	LumP	LumP	Luminal papillary	Luminal papillary	neg	pos	NOS
17	Ba/Sq	N/A	Basal squamous	N/A	pos	pos	NOS
9	Ba/Sq	Ba/Sq	Basal squamous	Basal squamous	pos	N/A	Squamous
46	Stroma-rich	LumNS	Luminal infiltrated	Luminal	neg	pos	NOS
20	LumNS	Stroma-rich	Luminal infiltrated	Lumina infiltrated	neg	pos	NOS

*IHC* Immunohistochemistry, *pos* positive (at least 10% of tumor cells show clear protein expression), *neg* negative, *NOS* not otherwise specified, *N/A* not available for evaluation

### Reduction of gene set for molecular subtyping

We used two previously described gene sets, that were developed for molecular subtyping of MIBC, named ESSEN1 and ESSEN2 in the present study [16, 20].

With HTP data, samples were classified as Ba/Sq in 6 (42.8%) and 7 (50%), LumP in 3 (21.4%) and 2 (14.3%), LumU in 1 (7.1%) and 1 (7.1%), LumNS in 0 and 1 (7.1%), NE-like in 1 (7.1%) and 1 (7.1%), stroma-rich in 3 (21.4%) and 2 (14.3%) cases using ESSEN1 and ESSEN2 gene sets, respectively. Using MACE data, samples were classified as Ba/Sq in 5 (35.7%) and 8 (57.1%), LumP in 2 (14.3%) and 1 (7.1%), LumU in 1 (7.1%) and 2 (14.3%), LumNS in 1 (7.1%) and 2 (14.3%), NE-like in 1 (7.1%) and 0 and stroma-rich in 3 (21.4%) and 2 (14.3%) cases using ESSEN1 and ESSEN2 gene sets, respectively (Figure S3).

Compared to the comprehensive transcriptomic data, the reduction in gene sets resulted in a significant increase of the correlation *p*-values associated to the correlation of the samples with the nearest centroids (Figure S4). The median correlation *p*-value was still < 0.0001. One sample (44) could not be classified with the MACE data and was excluded for the further analyses because the correlation *p*-value was above the set threshold of 0.2. Of note, this sample showed a lymphoepithelial histological phenotype, and the heavy infiltration of immune cells might have hindered correct classification.

The Pearson’s correlations coefficient for samples and each consensus class stratified for the called consensus classes are included in the supplementary material.

**Fig. 3:**
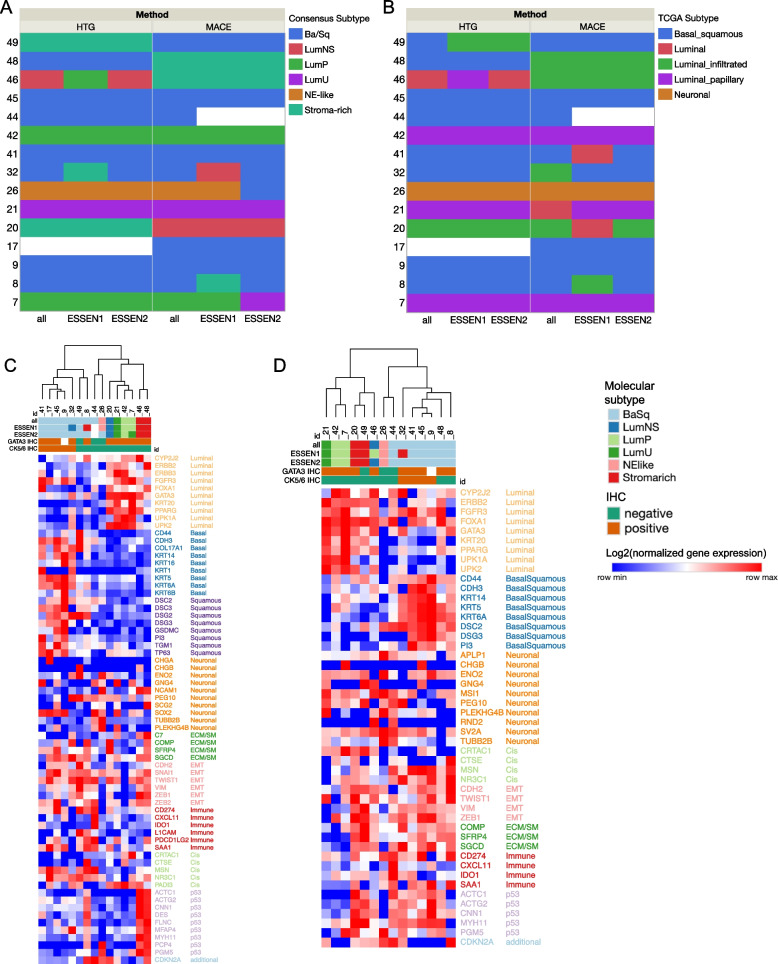
Heatmap showing the molecular subtypes according to the consensus classification (**A**) and the TCGA classification (**B**) for each sample as identified by all genes vs. different gene sets (all, ESSEN1, ESSEN2) and assay methods (HTP, MACE). **C**: Gene expression heatmap of MACE data and the ESSEN1 panel. **D**: Gene expression heatmap of the ESSEN2 panel with molecular consensus classes assigned using the HTP data. The heatmap was constructed with the open-source Morpheus software (https://software.broadinstitute.org/morpheus/) using log2-transformed normalized gene expression values. Hierarchical clustering was performed with Pearson’s correlation metric with complete linkage. Heatmaps also indicate the IHC-expression of CK5/6 and GATA3. Colors of genes correlate to the signature names. CIS: carcinoma in situ; ECM: extracellular matrix; EMT: epithelial-to-mesenchymal transition; SM: smooth muscle

For the ESSEN1 panel, consensus molecular subtypes were concordant using the HTP data in 86% (12/14). One sample (46) changed from LumNS to LumP and one sample (32) changed from Ba/Sq to stroma-rich. Using the MACE data, concordance was 86% (12/14). One sample (32) changed from Ba/Sq to LumNS and one sample (8) from Ba/Sq to stroma-rich (Fig. 3).

For the ESSEN2 panel concordance of the consensus molecular subtypes was 100% (14/14) with the HTP data (Fig. 3). Using the MACE data with the ESSEN2 panel, concordance was 86% (12/14). One sample (7) changed from LumP to LumU. Of note, one sample (26) with NE-like molecular subtype, that also showed neuroendocrine histological subtype and was negative for immunohistochemical CK5/6 and GATA3 expression, was misclassified by the ESSEN2 panel when using the consensus (but not TCGA classification) with the MACE (but not HTP) sequencing data as Ba/Sq. This sample also showed very low separation level when using all genes from the MACE data as input.

The overlap between subtypes according to the TCGA classification was 86% with the ESSEN1 and 93% with the ESSEN2 panel using the HTP data (Fig. 3B). With the MACE data the overlap was 86% (12/14) with the ESSEN2 panel and 64% with the ESSEN1 panel (two samples changed within luminal subtypes and three samples had discordant subtypes between basal/squamous and luminal subtypes).

## Discussion

Molecular analyses of bladder cancer specimens are emerging and provide elementary information to address bladder cancer research questions. One of the major goals of bladder cancer research is to identify subtypes with different sensitivity to systemic therapies such as chemotherapy, immune-checkpoint inhibition, and further targeted treatments. Although molecular subtype classification of bladder cancer has not yet been incorporated into therapeutic decision making, robust methods are important to achieve progress in clinical translation and validation and to improve reproducibility. FFPE samples represent snapshots of the histology and biological information of a tumor at the time of collection, while the patient is being treated and outcome data can be generated. It is important to use the information, that is always collected and stored as FFPE and to overcome limitations of degraded RNA.

In this study, we used FFPE-isolated RNA to determine molecular consensus subtypes using two different sequencing techniques. The overall agreement between molecular subtypes was high for both techniques, although different RNA, library preparation and sequencing facilities were used. We validated two reduced gene sets to determine molecular subtypes with high accuracy, that can be used as a panel-based method at lower cost, which is an important step to introduce subtyping into routine practice.

Efforts are being made to methodologically simplify subtype classification by using reduced gene sets to enable its implementation in clinical practice. Stratifying patients will be important to select patients, that respond to chemo- or immunotherapy to reduce unnecessary toxicities and costs, as soon as prospective and validated evidence is provided. In a previous study, a 47-gene panel (BASE47) was proposed for the discrimination between luminal and basal subtypes using the NanoString platform [14]. Recently, the ESSEN1 (*n* = 68) and ESSEN2 (*n* = 48) gene panels were developed to discriminate between 5–6 gene expression-based molecular subtypes according to different classification systems (e.g. TCGA, Consensus, Lund etc.) by using the qRT-PCR method on fresh frozen samples and the NanoString technique on FFPE samples, respectively [16, 20]. These reduced gene sets still allowed the classifier to designate molecular consensus molecular subtypes in all except for one case, which had a lymphoepithelial histological subtype. Our results show high accuracy of 85%-100% of both reduced panels to call consensus molecular subtypes, compared to the comprehensive transcriptomic data. Discordant subtypes were observed between the stroma-rich and the Ba/Sq or the LumNS subtype and between the different luminal subtypes. Divergent subtype calls can be the result of either differences in gene expression or the composition of genes used for calculation. According to our data, the reduced ESSEN2 panel showed an even higher overlap with the comprehensive transcriptome data than the ESSEN1 panel. Thus, the selection of genes might be more important for the classification than just the quantity.

So far as no use-case for the application of molecular subtypes exists, a comprehensive gene expression analysis provides additional information on further genes and enables the analysis of additional immune or stroma signatures, which could find application as predictive markers [30–32].

Limitations include the small number of samples. With only one sample for some of the subtypes general conclusions on which method is superior cannot be drawn. Furthermore, we did not perform a direct comparison between FFPE and fresh tissue. Our study lacks the comparison of HTP and MACE to further sequencing techniques used in previous molecular subtyping studies, like Illumina RNA seq or Affymetrix. Most importantly, sequencing was not performed with the exact same RNA, but RNA from neighboring tumor areas. Thus, discordant results can be the result of slight differences in tumor and stroma cell content or differences in deeper tissue layers not represented on the slide. However, this issue might reflect the heterogeneity of bladder cancer itself. This becomes particularly evident with the stroma-rich molecular subtype, since it was present in all divergent classified samples and highlights problems affecting bulk-RNA sequencing of bladder cancer samples in general. Molecular subtyping is performed on RNA derived from tissue (cores) of biopsy specimens representing only a fraction of the tumor. The subtype determined from the isolated RNA might not be the only and/or predominant subtype of a tumor, which is known to be heterogenous, especially in bladder cancer [17, 33]. Furthermore, the subtype assigned depends on the cellular components, of which the RNA is extracted and the proportion of stromal content will influence, if a tumor is called as stroma-rich or infiltrated [17, 31, 34, 35].

## Conclusion

The consensus molecular subtypes represent a robust classification and can be determined based on comprehensive or selected FFPE transcriptome data. Using the data of matching pairs, the agreement of subtypes was high, but differences were observed when the stroma-rich molecular subtype was involved. Based on our results, it seems important to further unravel the heterogeneity of bladder cancer and the influence of stromal components on molecular subtypes to reduce sampling bias and allow more accurate assignment.

## Supplementary Information

**Additional file 1:Supplementary material. Table S1.** ESSEN1 gene set. **Table S2.** ESSEN2 gene set. **Figure S1.** Comparison of correlation *p*-value between for samples older and younger than five years for MACE and HTG. **Figure S2.** Pearson’s correlation between samples and each consensus class stratified for the different methods and for the called consensus class. **Figure S3.** Relative distribution of subtype calls with each method and gene panels. **Figure S4.** A: Comparison of *p*-values associated to the Pearson’s correlation of the samples with the nearest centroid for each method (HTG and MACE) for the ESSEN1 and ESSEN2 gene panels. **Figure S5.** Pearson’s correlation between samples and each consensus class stratified for the different gene sets.

## Data Availability

The transcriptomic dataset generated and analyzed during the current study in the are available in the GEO repository: GSE225376. Further data that support the findings of this study are available from the corresponding author upon reasonable request.

## Abbreviations

Ba/Sq: Basal/squamous
FFPE: Formalin-fixed, paraffin-embedded
HTG: HTG Molecular Diagnostics, Inc.
HTP: HTG transcriptome panel
IQR: Interquartile range
LumNS: Luminal nonspecified
LumP: Luminal papillary
MACE: Massive Analysis of cDNA Ends
MIBC: Muscle-invasive bladder cancer
NE-like: Neuroendocrine-like
RNA: Ribonucleic acid
RT-qPCR: Real-time quantitative polymerase chain reaction
TCGA: The Cancer Genome Atlas
UCT: University Cancer Center Frankfurt
