# Supplementary material for: Optimizing identification of consensus molecular subtypes in muscle-invasive bladder cancer: a comparison of two sequencing methods and gene sets using FFPE specimens
Source: BMC Cancer. 2023 Jun 4;23:504. doi: 10.1186/s12885-023-11016-9 (PMC10239603; doi:10.1186/s12885-023-11016-9)
Supplement: Supplementary file 1 — Additional file 1:Supplementary material. Table S1. ESSEN1 gene set. Table S2. ESSEN2 gene set. Figure S1. Comparison of correlation p-value between for samples older and younger than five years for MACE and HTG. Figure S2. Pearson’s correlation between samples and each consensus class stratified for the different methods and for the called consensus class. Figure S3. Relative distribution of subtype calls with each method and gene panels. Figure S4. A: Comparison of p-values associated to the Pearson’s correlation of the samples with the nearest centroid for each method (HTG and MACE) for the ESSEN1 and ESSEN2 gene panels. Figure S5. Pearson’s correlation between samples and each consensus class stratified for the different gene sets. [file 12885_2023_11016_MOESM1_ESM.pdf]

Supplementary material

Optimizing identification of consensus molecular subtypes in muscle-invasive bladder cancer: A comparison of two sequencing methods and gene sets using FFPE specimens

Table S1: *ESSEN1 gene set [1]*

CYP2J2	KRT6A	TUBB2B	SAA1
ERBB2	KRT6B	PLEKHG4B	CRTAC1
ERBB3	DSC2	C7	CTSE
FGFR3	DSC3	COMP	MSN
FOXA1	DSG2	SFRP4	NR3C1
GATA3	DSG3	SGCD	PADI3
KRT20	GSDMC	CDH2	ACTC1
PPARG	PI3	SNAI1	ACTG2
UPK1A	TGM1	TWIST1	CNN1
UPK2	TP63	VIM	DES
CD44	CHGB	ZEB1	FLNC
CDH3	ENO2	ZEB2	MFAP4
COL17A1	GNG4	CD274	MYH11
KRT14	NCAM1	CXCL11	PCP4
KRT16	PEG10	IDO1	PGM5
KRT1	SCG2	L1CAM	CHGA
KRT5	SOX2	PDCD1LG2	CDKN2A

Table S2: *ESSEN2 gene set [2]*

CYP2J2	KRT5	RND2	SFRP4
ERBB2	KRT6A	SV2A	SGCD
FGFR3	DSC2	TUBB2B	CD274
FOXA1	DSG3	CRTAC1	CXCL11
GATA3	PI3	CTSE	IDO1
KRT20	APLP1	MSN	SAA1
PPARG	CHGB	NR3C1	ACTC1
UPK1A	ENO2	CDH2	ACTG2
UPK2	GNG4	TWIST1	CNN1
CD44	MSI1	VIM	MYH11
CDH3	PEG10	ZEB1	PGM5
KRT14	PLEKHG4B	COMP	CDKN2A

Figures

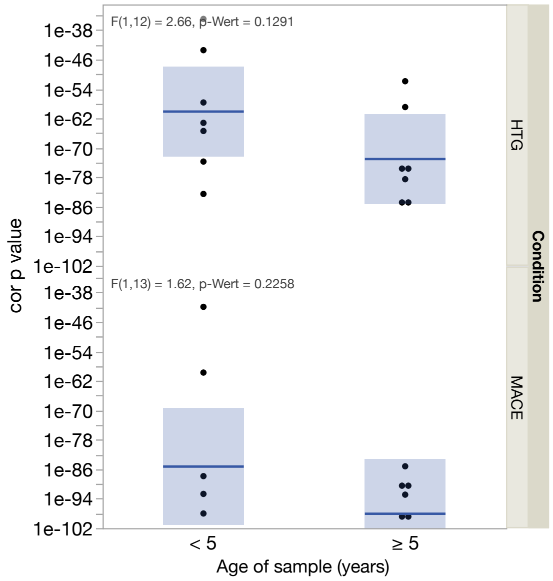

Figure S1: *Comparison of correlation p-value between for samples older and younger than five years for MACE and HTG. MACE: <5years: median 2e-93; ≥5years: median 6e-94; HTG: <5years: median 7e-64; ≥5years median 1e-76; p>0.1, respectively.*

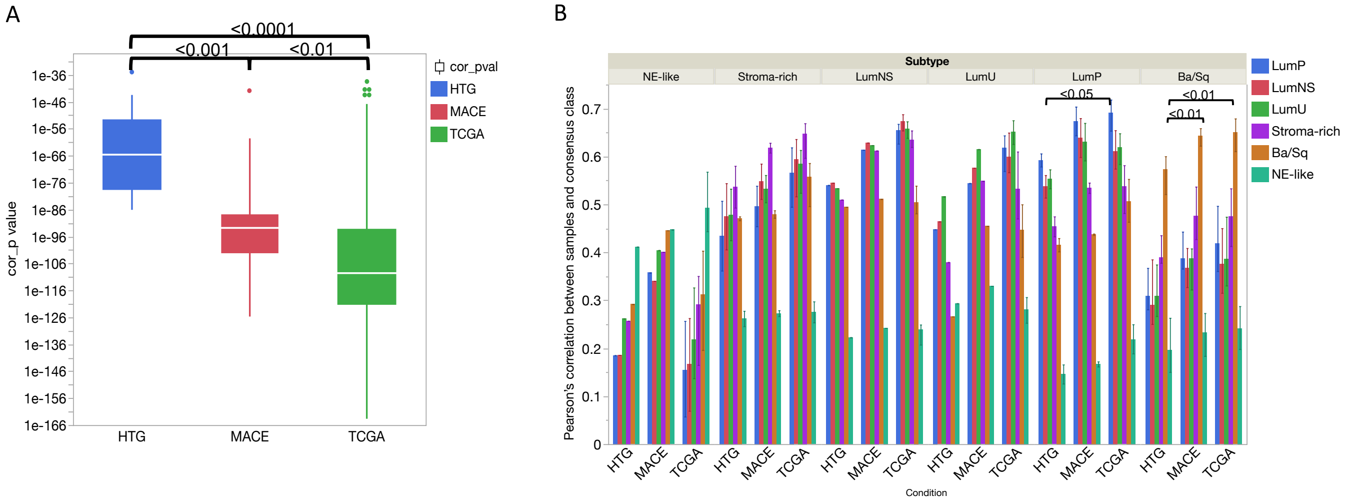

Figure S2: *A: Comparison of p-values associated to the Pearson's correlation of the samples with the nearest centroid (cor p_value) for each method (HTG and MACE) and to the TCGA data set. B: Pearson's correlation between samples and each consensus class stratified for the different methods and for the called consensus class.*

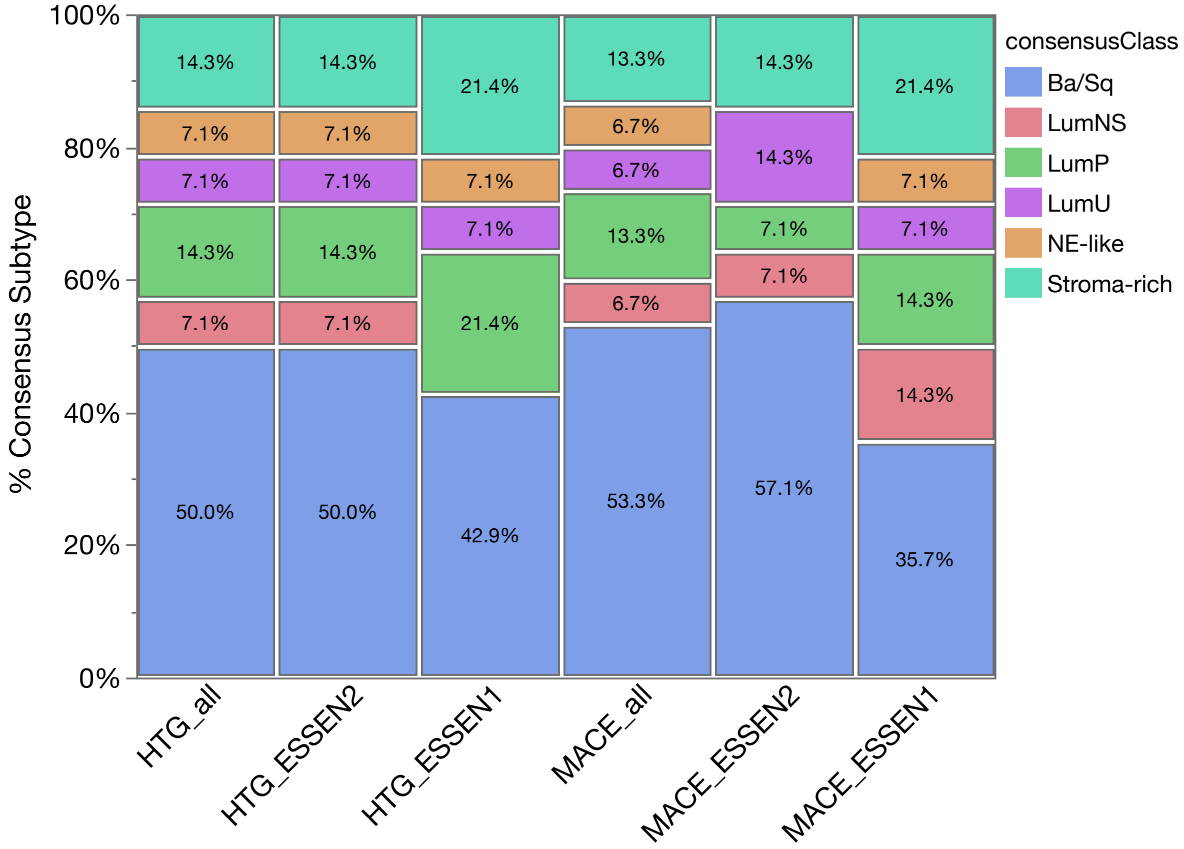

Figure S3: *Relative distribution of subtype calls with each method and gene panels*

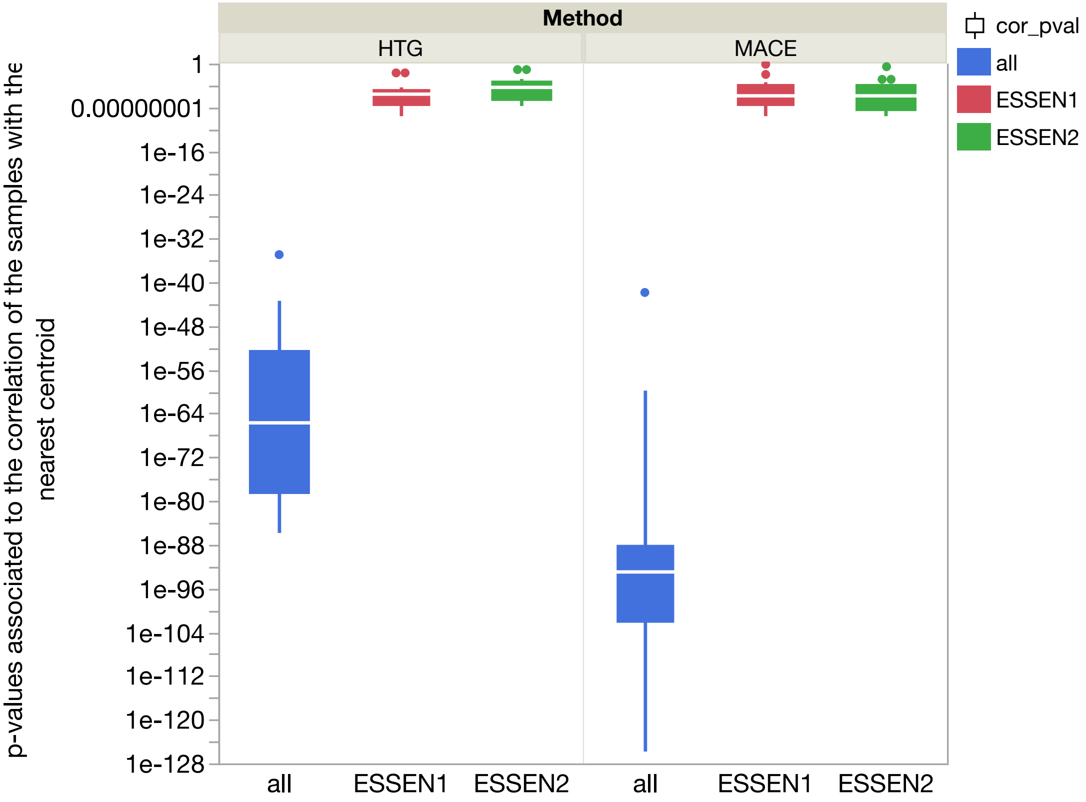

Figure S4: *A: Comparison of p-values associated to the Pearson's correlation of the samples with the nearest centroid for each method (HTG and MACE) for the ESSEN1 and ESSEN2 gene panels.*

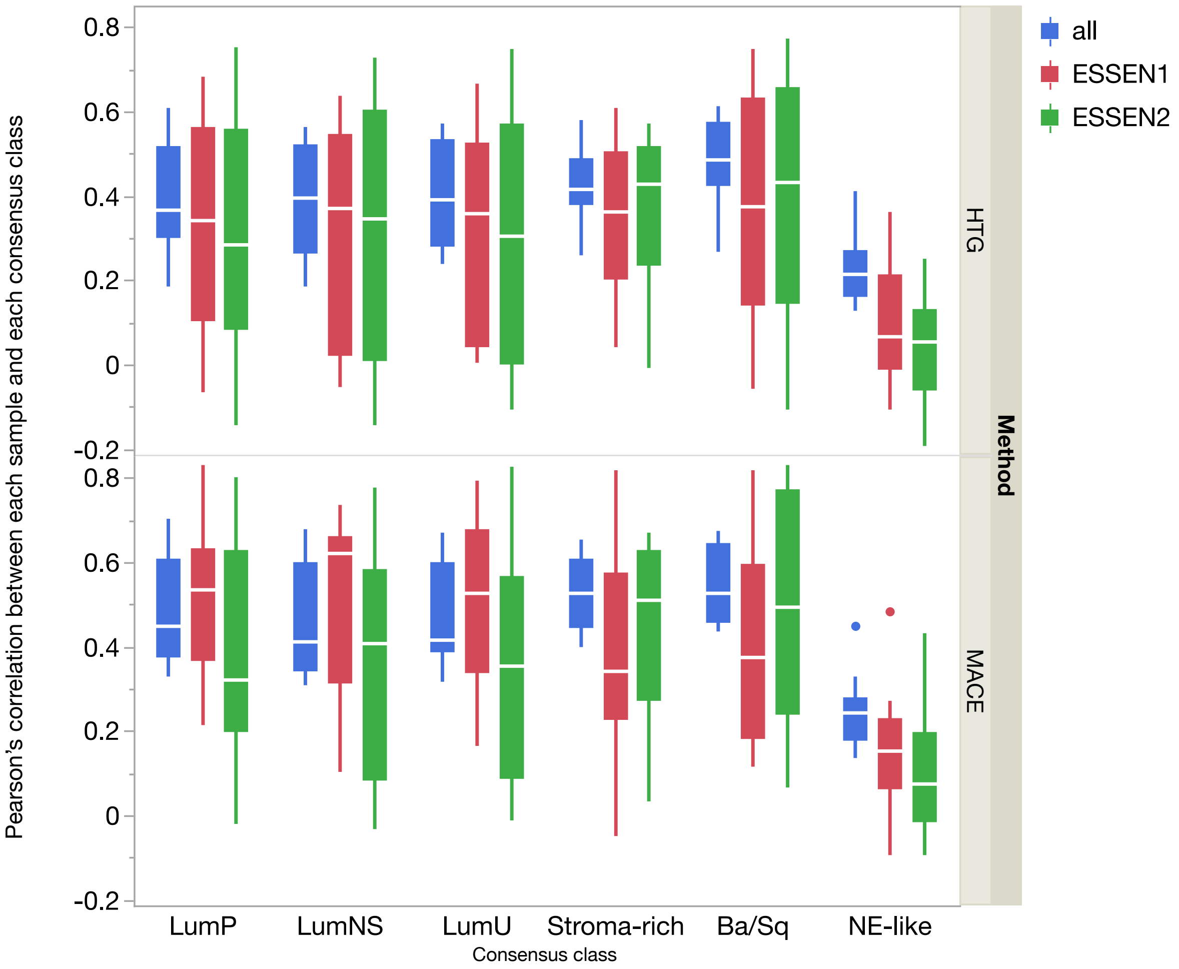

Figure S5: *Pearson's correlation between samples and each consensus class stratified for the different gene sets (comprehensive transcriptomic data = all; ESSEN1 and ESSEN2) and for the called consensus class.*

References

1. Olah C, Hahnen C, Nagy N, Musial J, Varadi M, Nyiro G, Gyorffy B, Hadaschik B, Rawitzer J, Ting S *et al*: **A quantitative polymerase chain reaction based method for molecular subtype classification of urinary bladder cancer-Stromal gene expressions show higher prognostic values than intrinsic tumor genes**. *International journal of cancer Journal international du cancer* 2021.
2. Olah C, Reis H, Hoffmann MJ, Mairinger F, Ting S, Hadaschik B, Krafft U, Grunwald V, Nyirady P, Varadi M *et al*: **Predictive value of molecular subtypes and APOBEC3G for adjuvant chemotherapy in urothelial bladder cancer**. *Cancer Med* 2022.
